# The Proteome of the Differentiating Mesencephalic Progenitor Cell Line CSM14.1 *In Vitro*

**DOI:** 10.1155/2014/351821

**Published:** 2014-01-30

**Authors:** B. Weiss, S. Haas, G. Lessner, S. Mikkat, M. Kreutzer, M. O. Glocker, A. Wree, O. Schmitt

**Affiliations:** ^1^Department of Anatomy, University of Rostock, Gertrudenstraße 9, 18057 Rostock, Germany; ^2^Department of Pathology, University of Würzburg, Josef-Schneider Straße 2, 97080 Würzburg, Germany; ^3^Proteome Center Rostock, University of Rostock, Schillingallee 69, 18055 Rostock, Germany

## Abstract

The treatment of Parkinson's disease by transplantation of dopaminergic (DA) neurons from human embryonic mesencephalic tissue is a promising approach. However, the origin of these cells causes major problems: availability and standardization of the graft. Therefore, the generation of unlimited numbers of DA neurons from various types of stem or progenitor cells has been brought into focus. A source for DA neurons might be conditionally immortalized progenitor cells. The temperature-sensitive immortalized cell line CSM14.1 derived from the mesencephalon of an embryonic rat has been used successfully for transplantation experiments. This cell line was analyzed by unbiased stereology of cell type specific marker proteins and 2D-gel electrophoresis followed by mass spectrometry to characterize the differentially expressed proteome. Undifferentiated CSM14.1 cells only expressed the stem cell marker nestin, whereas differentiated cells expressed GFAP or NeuN and tyrosine hydroxylase. An increase of the latter cells during differentiation could be shown. By using proteomics an explanation on the protein level was found for the observed changes in cell morphology during differentiation, when CSM14.1 cells possessed the morphology of multipolar neurons. The results obtained in this study confirm the suitability of CSM14.1 cells as an *in vitro* model for the study of neuronal and dopaminergic differentiation in rats.

## 1. Introduction

The motoric cardinal symptoms (rigor, tremor, akinesia, and postural instability) in Parkinson's disease (PD) are caused by the degeneration of dopaminergic (DA) neurons. Most of these dopaminergic neurons are located in the substantia nigra pars compacta. The classical, symptomatic treatment of the disease includes the use of pharmaceuticals like L-DOPA or the more invasive deep brain stimulation. Furthermore, over the last three decades the concept of cell replacement has been brought into focus. In various clinical trials postmitotic DA neurons from human embryonic mesencephalic tissue have demonstrated to be the most prospective cells for transplantation in human PD brains [1, 2].

However, the origin of these cells from human embryos causes their major limitation concerning tissue availability and standardization of the graft. Therefore, to establish cell replacement therapy as an available therapeutic option for many PD patients, other ways to generate DA neurons in unlimited number and consistent quality have to be found. Over the last years various protocols for the production of DA neurons, for example, from embryonic stem cells or foetal neuronal stem cells, have been used. Another approach is the generation of DA neurons via induced pluripotent stem cells [3]. However, the use of conditionally immortalized progenitor cells is also a promising approach due to nearly unlimited access of material [4].

The temperature-sensitive immortalized mesencephalic progenitor cell line CSM14.1 derived from a 14-day-old rat embryo [5–8] differentiates *in vitro* in tyrosine hydroxylase (TH) and aldehyde-dehydrogenase-2 (ALD2)-expressing neurons. Undifferentiated CSM14.1 cells also contain the stem cell marker nestin and also the expression of Nurr-1—a member of the superfamily of orphan nuclear retinoic acid receptors—which plays an important role in the differentiation of dopaminergic neurons, has been described [9]. During differentiation the cells also show a change from an epithelial fibroblast-like phenotype to a morphology resembling multipolar neurons. After transplantation into the striatum of neonatal hemiparkinsonian rats the differentiation into TH-expressing cells and an improvement in motoric function could be demonstrated [10].

In contrast to the above mentioned results concerning the characterization of CSM14.1 cells *in vitro* obtained by using immunocytochemistry and western blotting, by the use of proteomic approaches important issues such as protein amount, protein stability, subcellular localization of proteins, posttranslational modifications, and protein-protein interactions can be elucidated [11]. Therefore, in this study we investigated the ability of the cell line CSM14.1 to function as a model for the neuronal and dopaminergic differentiation in rats by combining unbiased stereological evaluation of cell type specific marker proteins with 2D-gel electrophoresis followed by mass spectroscopy to analyze the differentially expressed proteome.

## 2. Material and Methods

### 2.1. Cell Culture and Immunocytochemistry

Immortalized CSM14.1 cells [5] were cultivated and expanded as described by Haas and Wree [9] in DMEM supplemented with 10% fetal calf serum (FCS), 100 Units mL^−1^ penicillin, and 100 *μ*g mL^−1^ streptomycin in a humidified incubator (95% air, 5% CO_2_, 33°C). Cell passage was done every third day. To induce differentiation the amount of FCS was reduced to 1% and the temperature was risen to 39°C—nonpermissive temperature [12, 13]. The media was routinely changed every third day. All cell tissue reagents were obtained from Gibco Invitrogen Corporation, Carlsbad, CA, USA.

### 2.2. For Immunocytochemistry CSM14.1 Cells Were Cultivated in 24 Well Plates

Undifferentiated cells and cells after 14 days and 28 days of differentiation, respectively, (see above) were washed with 0.1 M PBS (pH 7.4) and fixed in 3.7% paraformaldehyde solution (solved in 0.1 M PBS, pH 7.4) for a minimum of one hour. After three washes with PBS (pH 7.4) the cells were preincubated for 60 minutes in PBS (pH 7.4) containing 3% bovine serum albumine (BSA), 0.025% Triton X-100, and 3% normal horse serum (NHS) to block unspecific binding sites.

Incubation with the primary antibodies directed against the neural stem cell protein (nestin, mouse monoclonal, 1:500, BD Biosciences, San Jose, CA, USA), glial fibrillary acidic protein (GFAP, mouse monoclonal, 1:400, Sigma-Aldrich Corporation, St. Louis, MO, USA), neuronal nuclei antigen (NeuN, mouse monoclonal, 1:1000, Chemicon, Billerica, MA, USA), and tyrosine hydroxylase (TH clone 2, mouse monoclonal, 1:500, Sigma-Aldrich) dissolved in 0.1 M PBS containing 0.025% Triton X-100 and 1% BSA was done at 4°C overnight. For each time point and antibody four independent experiments were performed. After washing for three times with PBS (pH 7.4) the cells were incubated with the Cy3-conjugated secondary antibody (Donkey antimouse IgG + IgM, 1:500, Jackson ImmunoResearch Laboratories, Inc., West Grove, PA, USA) dissolved similar to primary antibodies at 4°C overnight. For cell counting the cell nuclei were stained with 4.6-diamidino-2-phenylindol dihydrocloride (DAPI, Carl Roth GmbH + Co.KG, Karlsruhe, Germany).

For the various cell type specific markers four different culture wells per marker were examined for each of the three different groups, leading to the examination of 48 cell culture wells.

### 2.3. Cell Counting and Statistics

Microphotography and cell counting were performed with an Olympus BX 51 microscope and the Stereo Investigator v8.0 (MicroBrightField Bioscience, Vermont, USA) software. Cells were counted using the 10x objective and an unbiased counting frame [14]. A characteristic point of a cell was applied to decide if the cell should be counted. Hereby the cell nuclei were chosen. Quantification was performed in region of the whole cell culture well that was placed under the circular cover slip (diameter 1.2 cm). Counting frames had a dimension of 500 × 500 *μ*m² and a distance to each other of 1000 *μ*m. A systematic random sampling, controlled by the Stereo Investigator software, ensured that frame regions were not double counted. An average of about 500 cells per culture well were examined during the counting procedure. The Chi-Quadrat test and Fisher's exact test were used (SPSS v11.01, SPSS Inc. IBM Company Headquarters, Chicago, IL, USA) to compare cell counts.

### 2.4. Proteomics

For proteomics CSM14.1 cells were cultivated and differentiated in tissue culture dishes as described above. After removal of the culture medium undifferentiated cells (day 0) and cells after 28 days of differentiation (day 28) were washed twice with ice cold PBS (pH 7.4). Afterwards the cells were mechanically removed from the bottom of the tissue culture dishes in 1 mL ice cold PBS each. The cell suspension was fractionated in 1.5 mL reaction tubes which underwent centrifugation for 5 min at 4°C and 5000 rpm (Heraeus Megafuge 1.0R, Thermo Fisher Scientific Inc., Waltham, MA, USA, Rotor 3041). The remaining mass of each cell pellet was approximately 150 mg. After freezing at −80°C (9 × probe mass (mg)) *μ*L lysis buffer (containing 7 M urea (Sigma-Aldrich), 2 M thiourea (Sigma-Aldrich), 70 mM DTT (Sigma-Aldrich), 4% w/v CHAPS (Sigma-Aldrich), 0.5% ampholyte high resolution ph 3–10 (Sigma-Aldrich)), (0.4 × mass probe (mg)) *μ*L Complete (Roche Diagnostics GmbH, Basel, Swiss), (0.1 × probe mass (mg)) *μ*L PMSF (Sigma-Aldrich), and (0.1 × probe mass (mg)) *μ*L PepA (Sigma-Aldrich) were added. The tubes were quickly frozen in liquid nitrogen, warmed up at room temperature, sonificated in an ice cold ultrasound bath for 5 min and then centrifuged for 20 min at 4°C and 15.000 rpm (Megafuge 1.0R, Rotor 3041). Protein concentration was measured using a Bradford solution by Sigma-Aldrich. Sample aliquots were stored at −80°C.

The isoelectric focussing (IEF) procedure was performed with 18 cm nonlinear Immobiline DryStrip pH 3–10 (GE Healthcare Bio-Sciences AB, Uppsala, Sweden) which was rehydrated at 20°C for 20 h. Each strip was loaded with 500 *μ*g protein using cup-loading technology at anode and cathode. Electric focussing was performed with a Protean IEF Cell (Bio-Rad Laboratories, Inc., Hercules, CA, USA) at 8000 V resulting in approximately 100 kV h. Furthermore, IEF strips were incubated with equilibration buffer containing 1.5 M Tris-HCl pH 8.8, 6 M urea, 30% glycerol, 2% SDS, and 64.8 mM DDT for 30 min followed by a second incubation with the same buffer containing 20 mM 2-VP instead of DTT. Second dimension was carried out on 12% SDS gels using a Protean Plus Dodeca Cell (BioRad) at 125 V and 12°C for approximately 16 hrs. Afterwards gels were fixed with 45% methanol and 1% acetic acid for 24 hrs. Staining was performed using Brilliant-Blue Coomassie G250 (Carl Roth). For digitization at 300 dpi/12 bit a Heidelberg Nexscan F4100 (Heidelberger Druckmaschinen, Heidelberg, Germany) was used. For further analysis the program Progenesis PG 200 version 2006 (Nonlinear Dynamics Ltd., Newcastle, Great Britain) was applied.

Image analysis was performed using Progenesis PG200 Version 2006 (Nonlinear Dynamics Ltd., Newcastle upon Tyne, U.K.). Two groups of experimental gels (6 gels from day 0 and 6 gels from day 28) were registered to a reference gel chosen from day 0 gels. Spots showing a 2.5-fold larger or lower spot volume in 5 or 6 different gels of each group were considered up- or downregulated. Absent spots were defined as spots found in 5 or 6 gels of the day 28 group and not found in any gel of the day 0 group and the reference gel.

Spot picking, in-gel enzymatic digestion of proteins, and MALDI-TOF-MS analysis were performed as described circumstantially in Lessner et al. [15]. For protein identification the UniProtKB/Swiss-Prot database was used.

## 3. Results

### 3.1. Immunocytochemistry and Cell Counting

**Figure 1:**
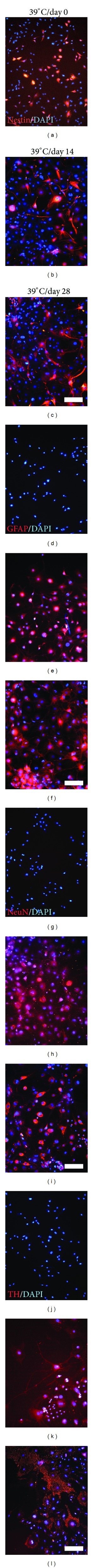
Results from ICC-staining of CSM14.1 cells during differentiation are shown. Images do not represent counting frame pictures and the numbers and distribution of immunoreactive cells should not be compared with the stereological results. However, morphological changes and different contents of cell type specific markers are recognizable. In the first row ((a), (b), (c)) images of staining against nestin (red) and DAPI (blue) are merged. A significant decrease of nestin-expression between undifferentiated cells (a) and cells that differentiated for 14 days (b) or 28 days (c) could be observed. In the second row ((d), (e), (f)) images of staining against GFAP (red) and DAPI (blue) are merged. Undifferentiated cells do not express GFAP (d). After 14 days of differentiation GFAP-positive cells could be detected (d) and an increase of positive cells could be shown after 28 days of differentiation (f). In the third row ((g), (h), (i)) images of staining against the neuronal marker NeuN (red) and DAPI (blue) are merged. No NeuN-positive cells could be detected in undifferentiated cells (g), whereas NeuN expressing cells could be found after 14 days of differentiation (h) and an increase in NeuN-positive cells could be observed after 28 days of differentiation (i). In the fourth row ((j), (k), (l)) images of staining against TH (red) and DAPI (blue) are merged. Undifferentiated cells do not express TH (j). TH-positive cells could be found after 14 days of differentiation (k) and an increase of TH-expressing cells was observed after 28 days of differentiation (l). Scale bars = 200 *μ*m ((a)–(l)).

**Figure 2:**
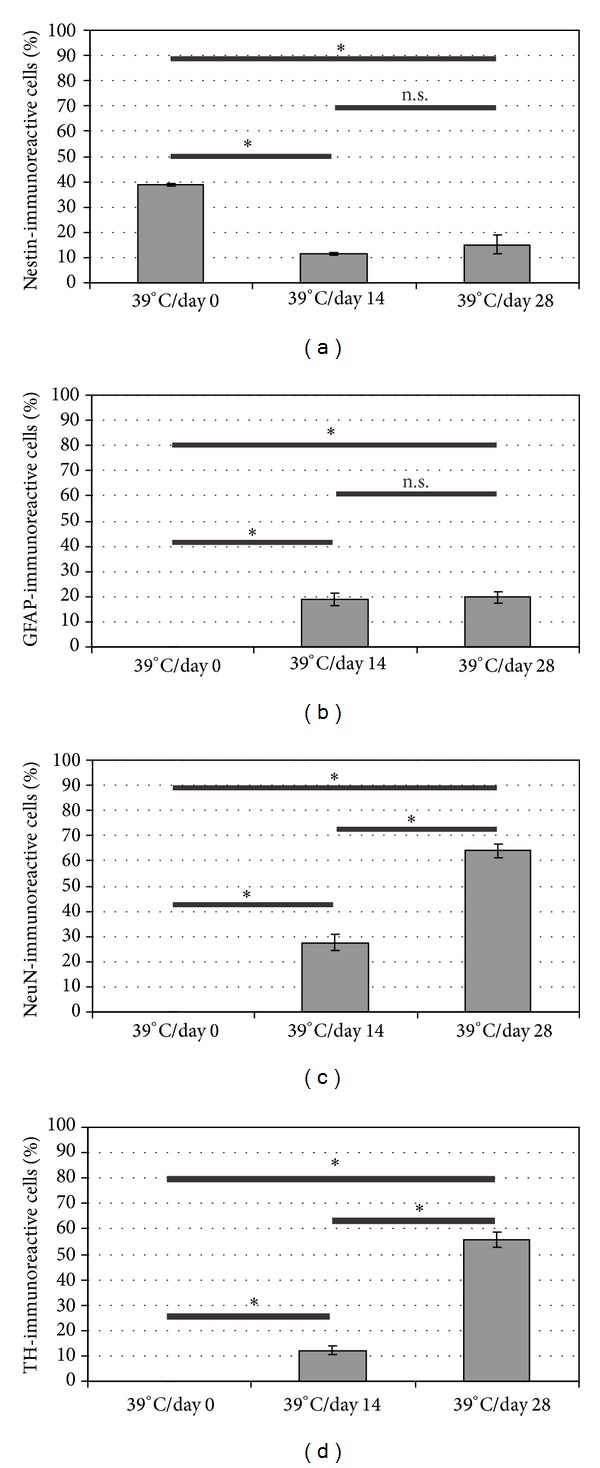
Results from unbiased cell counting are shown. In undifferentiated cells an amount of 38.74% (±0.62) nestin-positive cells (a) was found. During differentiation a significant decrease in the amount of positive cells could be observed. After 14 days 11.46% (±0.53) and after 28 days 15.09% (±3.72) of all cells were nestin-positive. GFAP (b) could not be detected in undifferentiated cells. After 14 days of differentiation 18.72% (±2.54) of all cells were GFAP-immunoreactive. The amount of GFAP-immunoreactive cells did not change significantly after 28 days of differentiation up to 19.66% (±2.04). Undifferentiated cells did not contain NeuN (c). After 14 days of differentiation 27.56% (±3.31) were NeuN-positive and a significant increase up to 64.06% (±2.74) could be observed after 28 days of differentiation. TH (d) could not be found in undifferentiated cells, but a significant increase in the amount of TH-immunoreactive cells from 12.07% (±1.71) after 14 days up to 55.69% (±2.92) after 28 days of differentiation could be observed. Error bars show SEM. *Significant difference from the respective time point; *P* < 0.001. For statistical analysis Chi-Quadrat test and Fisher's exact test were applied.

The type VI intermediate filament (IF) protein nestin is a widely-used marker for neuronal progenitor cells. As compared to previous studies [9, 16], here we also demonstrate that a large portion of undifferentiated CSM14.1 cells was immunoreactive for nestin (Figure 1(a)) and that during differentiation the number of nestin-immunoreactive cells decreased (Figures 1(b), 1(c), and 2(a)). The results of unbiased stereological cell counting revealed a significant decrease of nestin-containing cells from 38.74% (±0.62) at day zero to 11.46% (±0.53) at 14 days of differentiation (*P* < 0.001). The number of nestin-immunoreactive cells after 28 days of differentiation was 15.09% (±3.72) (Figure 2(a)) and was significantly lower than at day zero (*P* < 0.001) but did not significantly differ from day 14 (Figure 2).

GFAP, a member of type III IF proteins, is known as an important and obligatory protein of astrocytes [17]. Undifferentiated CSM14.1 cells were not immunoreactive for GFAP in this study (Figure 1(d)), whereas after 14 and 28 days of differentiation, respectively, an increase in GFAP-immunoreactivity was observed (Figures 1(e) and 1(f)). The amount of GFAP-containing cells 14 days after differentiation was 18.72% (±2.54) and this number increased to 19.66% (±2.04) 28 days after differentiation (Figure 2(b)). At both time points of differentiation the number of GFAP-containing cells was significantly different (*P* < 0.001) from the starting point but not significantly different between 14 and 28 days of differentiation. However, an increase in fluorescence intensity in GFAP-immunoreactive cells over time was observed indicating higher contents of GFAP after 28 days of differentiation.

A commonly used marker for postmitotic nerve cells is the neuronal nuclear protein NeuN with unknown function. In undifferentiated CSM14.1 cells the expression of NeuN could not be detected (Figure 1(g)) but its content in the CSM14.1 cells increased constantly during differentiation (Figures 1(h) and 1(i)). After 14 days of differentiation the number of 27.56% (±3.31) of cells was immunoreactive for NeuN (Figure 2(c)) and a further increase up to 64.06% (±2.74) after 28 days of differentiation could be shown (Figures 1(i) and 2(c)). At both differentiation time points the NeuN-immunoreactive cell numbers were significantly higher compared to the starting point (*P* < 0.001) and the increase between 14 and 28 days of differentiation was also significantly different (*P* < 0.001).

A similar result was also found for TH, the pacemaker enzyme of dopamine biosynthesis, which is a widely used protein to identify dopaminergic neurons. Undifferentiated CSM14.1 cells did not show any immunoreactivity for TH (Figure 1(j)). During differentiation TH was detectable after 14 days (Figure 1(k)) and the number of TH-containing cells increased after 28 days (Figure 1(l)). Like for the GFAP immunofluorescence signal, the TH containing cells seemed to contain more of the epitope due to an increase of fluorescence intensity. After 14 days of cultivation at 39°C 12.07% (±1.71) of the cells were TH-positive and a significant increase up to 55.69% (±2.92) after 28 days of differentiation was detected (Figure 2(d)). At both differentiation time points the TH-immunoreactive cell numbers were significantly higher compared to the starting point (*P* < 0.001) and the increase between 14 and 28 days of differentiation was also significantly different (*P* < 0.001).

### 3.2. Proteomics

**Figure 3:**
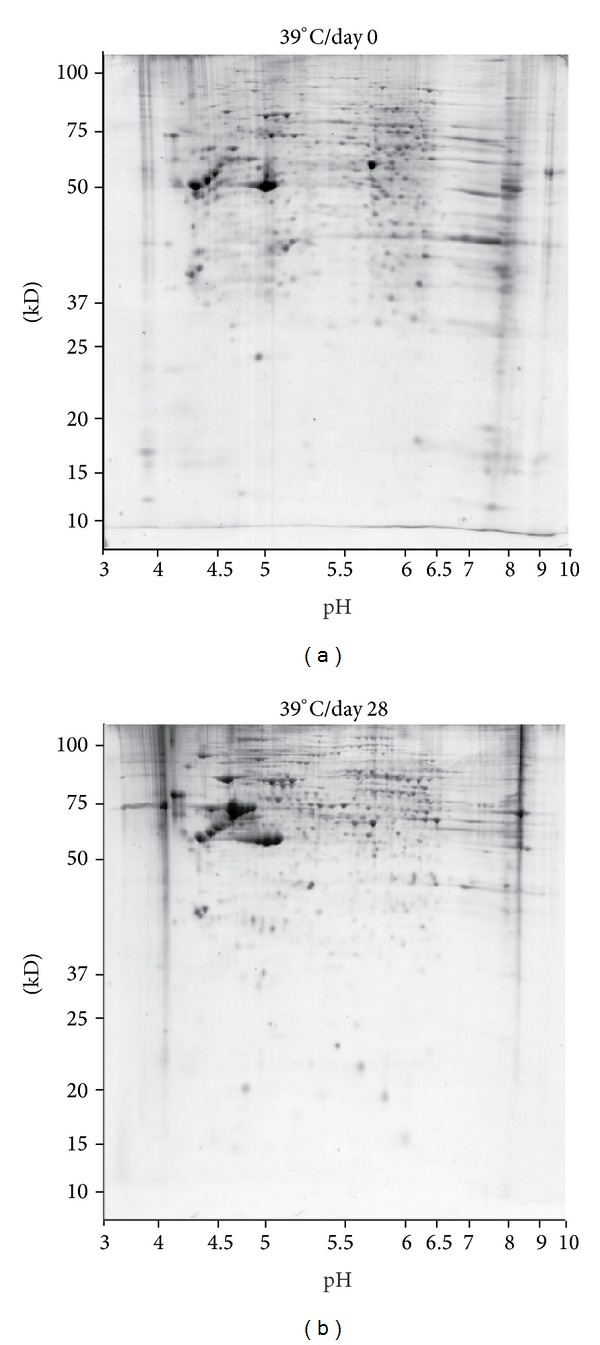
Comparison of 2 DE images of undifferentiated CSM14.1 cells (a) and of CSM14.1 cells after 28 days of differentiation (b). For spot visualization Coomassie-staining was used. Two groups of experimental gels (6 gels from day 0 and 6 gels from day 28) were registered to a reference gel chosen from day 0 gels. Spots showing a 2.5-fold larger or lower spot volume in 5 or 6 different gels of each group were considered up- or downregulated. Absent spots were defined as spots found in 5 or 6 gels of the day 28 group and not found in any gel of the day 0 group and the reference gel. In the reference gel 506 spots could be detected and 70.2% (±5.3) of the spots from the experimental gels day 0 could be matched onto the reference gel. In contrast, only 49.2% (±3.37) of the spots from the experimental gels day 28 found a match on the reference gel. Using the selection criteria as shown above, 27 spots were found upregulated in differentiated CSM14.1 cells, 24 spots downregulated, and 46 spots were detected as absent (i.e., only found in differentiated CSM14.1 cells). Via MALDI-TOF analysis 64 proteins could be identified.

**Figure 4:**
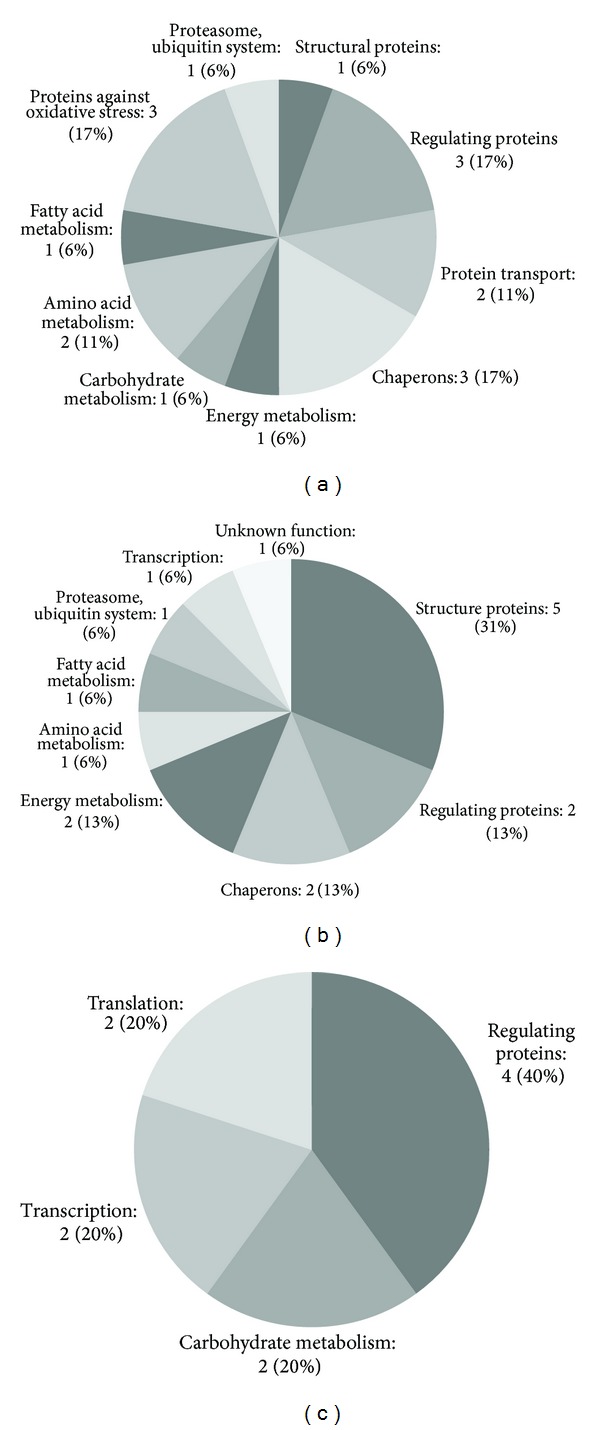
Relative quantitative distribution of functional protein groups only detected in differentiated CSM14.1 cells (a) and of proteins with a higher (b) or lower (c) expression compared to undifferentiated CSM14.1 cells. The majority of proteins only detected in differentiated CSM14.1-cells (a) were classified as regulating proteins (17%), chaperons (17%), and proteins against oxidative stress (17%). Upregulated proteins (b) belonged primarily to structural proteins (31%), regulating proteins (13%), chaperons (13%), and proteins of energy metabolism (13%). Proteins with a lower expression in differentiated CSM14.1 cells (c) were classified as regulating proteins (40%), proteins associated with transcription (20%) and translation (20%), and carbohydrate metabolism (20%).

In the reference gel 506 spots could be detected and 70.2% (±5.3) of the spots from the experimental gels at day 0 could be matched onto the reference gel (Figure 3(a)). In contrast, only 49.2% (±3.37) of the spots from the experimental gels at day 28 found a match on the reference gel (Figure 3(b)). Using the selection criteria as shown above, 27 spots were found upregulated in differentiated CSM14.1 cells, 24 spots downregulated, and 46 spots were detected as absent (i.e., only found in differentiated CSM14.1 cells). Via MALDI-TOF MS analysis 64 proteins could be identified (Table 1). The majority of proteins only detected in differentiated CSM14.1 cells (Figure 4(a)) were classified as regulating proteins (17%), chaperons (17%), and proteins against oxidative stress (17%). Upregulated proteins (Figure 4(b)) belonged primarily to structural proteins (31%), regulating proteins (13%), chaperons (13%), and proteins of energy metabolism (13%). Proteins with a lower expression in differentiated CSM14.1 cells (Figure 4(c)) were classified as regulating proteins (40%), proteins associated with transcription (20%) and translation (20%), and carbohydrate metabolism (20%).

## 4. Discussion

### 4.1. Immunocytochemistry and Cell Counting

As a parallel approach by characterising the changes in the proteome of differentiating CSM14.1 cells we used various cell type specific antibodies to document the morphological and phenotypical alterations over time. Hereby we also used unbiased stereology to quantify the differences in immunoreactive cell numbers. Undifferentiated CSM14.1 cells were known to express the stem cell marker nestin [9]. In this study we were able to show that only 38.74% (±0.62) of all cells are immunoreactive for nestin which is a contrary finding to the clonal origin of the cell line [5] and might be explained by proliferating cells in different phases of the cell cycle. After 28 days of differentiation 15.09% (±3.72) of all cells still express nestin which indicates that differentiation may not be complete in all cells at this time point [18]. In this study GFAP could not be detected via immunocytochemistry in undifferentiated CSM14.1 cells which is a contrary finding to Vernon and Griffin [16], who demonstrated immunoreactivity in western blots and immunocytochemistry by using polyclonal antibodies against GFAP. Moreover, Vernon and Griffin [16] showed, after an initial increase of GFAP after two weeks of differentiation, a constant decrease below the GFAP content of undifferentiated cells. Our findings, by using a monoclonal primary antibody directed against GFAP, indicate that in undifferentiated CSM14.1 cells GFAP is not detectable but that under differentiation conditions about 20 percent of the cells contain GFAP. This discrepancy between the findings of Vernon and Griffin and our recent results could be explained by the use of polyclonal antibodies. The use of polyclonal antibodies *in vivo* and also *in vitro* is problematic due to the cause of the unspecific cross reactivity of antibodies produced from the serum of immunized animals [19]. The increase of neuronal markers and TH in differentiating CSM14.1 cells is in line with our previous studies [9] and with findings from other groups [16, 20].

Vernon and Griffin [16] showed that differentiated CSM14.1 cells express the neuron-specific, soluble nuclear protein NeuN at a significantly higher degree than undifferentiated CSM14.1 cells. *In vivo* NeuN-positive CSM14.1 cells were detectable after transplantation into the striatum of neonatal rats [10] or into the substantia nigra of adult hemiparkinsonian rats [21]. Assuming that CSM14.1 cells at permissive culture conditions (33°C, 10% FCS) are undifferentiated, these cells should not show immunoreactivity against NeuN, which could be demonstrated in the present study. Moreover, after 14 days of differentiation 27.56% of all cells were NeuN-positive. After 28 days of differentiation, there was a significant increase in immunoreactivity for NeuN up to 64.06%. These results point to an increasing differentiation of CSM14.1 cells into neurons over the observed time period.

In differentiated CSM14.1 cells TH, the pacemaker enzyme of dopamine biosynthesis, could be detected by western blotting [9, 16] and Vernon and Griffin [16] also achieved this by immunocytochemistry using a polyclonal antibody. In both works, a weak immunoreactivity for TH was also found in undifferentiated CSM14.1 cells by immunocytochemistry and western blots. *In vivo* TH-positive CSM14.1 cells were only detected after intrastriatal transplantation in neonatal rats [10].

In this study undifferentiated CSM14.1 cells showed no immunoreactivity for TH. This result is congruent with the results of NeuN and GFAP as shown above. There is also a significant increase in the amount of TH immunoreactive cells over the observed time period (12.07% at day 14 and 55.69% at day 28). The differences between our recent findings and the observations made by Vernon and Griffin in 2005 [16] might be explained by the use of a monoclonal antibody in our study. The detection of TH alone does not characterize a cell as dopaminergic, because dopamine can also be metabolized to the catecholamines epinephrine and norepinephrine [22, 23]. However, the expression of the enzyme ALDH2 by differentiated CSM14.1 cells [9] makes a dopaminergic differentiation most likely, because ALDH2 could be used for the detection of differentiated dopaminergic cells [24–26]. Nevertheless, in the future the expression of the dopamine transporter in CSM14.1 cells or the content of dopamine itself in culture of differentiated CSM14.1 cells should be proofed.

### 4.2. Proteomics

In this study it was shown that the expression of the protein annexin A5 by the cell line CSM14.1 between day 0 (undifferentiated cells) and day 28 (differentiated cells) is upregulated. The same result has also been shown for the neuronal progenitor cell line ST14A derived from the striatum of a fetal rat [11]. Annexin A5 is a 35 kD protein and was first discovered as an anticoagulant-acting protein in blood vessels [27]. It is also called annexin-5, annexin V, or lipocortin V (UniProtKP/Swiss-Prot). The annexins are a superfamily of calcium ions- and phospholipid-binding proteins with highly conserved binding domains [28–30]. Annexin I, III, and V inhibit the activity of phospholipase A2 (PLA2) which acts as a key enzyme in inflammation and cytotoxicity in the CNS [31]. The activated PLA2 cleaves membrane phospholipids and leads to cell death [32]. Simultaneously precursor molecules of eicosanoids and platelet-activating factor (PAF) are released, which promote the production of reactive oxygen species. There are more than 27 isoforms of PLA2 in mammals which can be divided into four main groups [33]: cytosolic PLA2, secretory PLA2, calcium ion-independent PLA2, and PAF acetylhydrolases. Especially the soluble PLA2 seems to play a key role in neurodegenerative diseases such as Alzheimer's disease [34], multiple sclerosis [35, 36], PD [37, 38], and in the response to spinal cord injuries [39].

Furthermore, it could be shown that annexin V plays an important role as a regulator of apoptosis [40]. Apoptotic cells display phosphatidylserine on the outer side of the cell membrane, which serves as a signal for phagocytosis by macrophages [41]. However this process is not only limited solely to apoptotic cells but also occurs in the context of nonapoptotic cell death programs [42] as well as in the aging of red blood cells and the activation of platelets [43]. Healthy cells prevent the exposure of phosphatidylserine on their cell surface by energy-dependent processes. Annexin V binds in the presence of calcium ions with high affinity to the negatively charged phosphatidylserine [40]. Annexin A5 is therefore used widely as a marker for the study of apoptosis *in vitro*, in animal models, and even *in vivo* in patients with cardiovascular disease or cancer.

Annexins I and V were also found to have neurotrophic effects on cultured neurons [44, 45]. Han et al. [46] studied the effects of the annexins II and V on the survival of neurons and astrocytes *in vitro*. They could show that these two proteins are essential for the survival and the growth of neurites of developing cortical neurons, for the survival of glial cells, and for the protection of neurons and glial cells against peroxides and hypoxic injury. Whether this observation is related to the inhibition of PLA2 by annexin V has to be clarified.

In the present study, the absolute number of CSM14.1 cells per petri dish is reduced by more than half during differentiation (see also Haas et al. [21]). The observed increase in the expression of annexin A5 during differentiation indicates that cell death might occur by apoptosis. However, this increase might also be explained by the previously described effect of annexin A5 for the development and survival of neurons and glia cells. The cell line CSM14.1 seems to be able to respond to an elevated level of cell stress due to the change of the environment with an increased expression of annexin A5.

Another protein with increased expression in differentiated CSM14.1 cells is cytoplasmic actin, a globular protein (G-actin), which forms in the presence of magnesium and calcium ions a microtubule independent cytoskeleton and is a fundamental part of the contractile apparatus of muscle cells. It also occurs in high concentrations in nonmuscle cells [47]. There is a dynamic equilibrium between monomeric and polymeric (filamentous) actin (F-actin). The polymerization is carried out at the positive end of a actin filament by addition of ATP complexed G-actin and the cleavage of G-actin occurs from the minus end. The structure of the actin filament was described by Holmes et al. [48]. Several proteins regulate the dynamic equilibrium through the stabilization of F-actin or the promotion of G-actin cleavage. One of these proteins is gelsolin (86 kD) which shows in this study an increased expression in differentiated CSM14.1 cells. It was first isolated in macrophages from the rabbit lung [49] and plays an important role in actin-based cell motility.

Depending on calcium ions gelsolin prevents further actin polymerization by covering the plus ends [49–53]. On the other hand it may encourage the formation of filaments by binding two monomers and therefore functions as a nucleus. The binding of phosphatidylinositol 4-phosphate or phosphatidylinositol-4,5-bisphosphate to gelsolin solves its binding to the plus end of the actin filament, so that a quick attachment of further monomers to this filament is possible [54, 55]. Gelsolin also plays an important role in the formation of neurites by regulation of actin polymerisation. PC12 cells that overexpress gelsolin develop longer neurites with a greater motility than PC12 wild type cells [56].

Gelsolin seems to ensure the stability of actin filaments [57]. Dong et al. showed that after entorhinal deafferentation of the hippocampus a significant increase of gelsolin expression could be observed in activated microglia and astrocytes [58]. Another protein with increased expression in differentiated CSM14.1 cells is the 48 kD actin related protein 3 (Arp3). A complex of Arp3 and actin related protein 2 (Arp2/3) allows the formation of branched actin filaments [59].

Physiologically associated with actin is the 68 kD protein moesin (membrane-organizing extension spike protein), which is detectable only in differentiated CSM14.1 cells. Moesin belongs together with ezrin and radixin to the family of ERM (ezrin-radixin-moesin) proteins which are highly conserved during evolution [60]. The ERM proteins play an important role in the formation and maintenance of cell shape within growth and motility of cells [61, 62]. The C-terminal domain of these proteins binds to actin [63–66]. The N-terminal domain, called the FERM (band 4.1, ezrin, radixin, moesin homology domain [67]) binds to the cytoplasmic domain of numerous integral membrane proteins. By intramolecular combination of the N- and C-terminus an inactive conformation is created, which prevents the binding of other proteins, such as F-actin [64]. At the C-terminus, there is a conserved threonine residue whose phosphorylation results in a conformational change and allows the association with other proteins [68]. This phosphorylation is executed, for example, by the Rho kinase [69]. The ratio between phosphorylated (pERM) and nonphosphorylated ERM proteins is important for the formation of neurites by neurons. In addition to the Rho kinase the LRRK2 (leucine-rich repeat protein kinase 2) is of great importance. Mutations in this enzyme are the cause of autosomal dominant forms of PD [70, 71]. This kinase is closely related to the preservation and the growth of neurites and therefore important for the development of neurons [72]. It is assumed that the formation of an axon from a neurite occurs when the stability of the F-actin decreases and the stability of microtubules increases [73, 74].

Taken together, proteins associated with the cytoskeleton which are necessary for morphological differentiation (cell processes) as well as migration *in vivo* after intracerebral transplantation [10, 21] are correlated with an increase of their differential expression. In further experiments protein validation and interactions could be performed with regard to the proteins detected here.

## 5. Conclusion

The changes in the expression pattern of the proteins discussed above are consistent with the previous findings for the cell line CSM14.1. The increased expression of actin, gelsolin, and Arp3 by differentiated cells and the expression of moesin only by differentiated CSM14.1 might be related and could be evidence for a neuronal development of these cells. These findings are in agreement with the morphological change of the cells during differentiation.

The detection of the proteins nestin (dimer: 198–260 kDa), GFAP (50 kD), NeuN (46–48 kD), and TH (60–68 kD) was not achieved with the selected differential experimental setting. Thus, a direct comparison of the results of both methods used in this study is not possible. Further investigation should include a protein mapping of undifferentiated and differentiated CSM14.1 cells to analyze spots which did not fulfill our established criteria. A fractional analysis of the individual cell compartments might also be a promising approach. Especially integrated membrane proteins might be a promising research objective in terms of neuronal and especially dopaminergic differentiation.

The results obtained in this study confirm the suitability of the cell line CSM14.1 as a model for the study of neuronal and dopaminergic differentiation in rats.

## Figures and Tables

**Table 1:** Differentially expressed proteins in CSM14.1 cells after 28 days of differentiation.

Acc. No.	Entry name	Protein name	Expression	Score	MW	pI	Qm	Sc	Mixed
Structural proteins									
Q6AYZ1	TBA1C_RAT	Tubulin alpha 1C-chain	Absent	56	50590	4.96	4	11	−
P31000	VIME_RAT	Vimentin	Up	205	53757	5.06	24	36	−
P70615	LMNB1_RAT	Lamin-B1	Up	188	66794	5.16	21	33	−
**P60711 **	**ACTB_RAT**	**Actin, cytoplasmic 1**	**Up**	**110**	**42052**	**5.29**	**9**	**25**	**−**
**Q4V7C7**	**ARP3_RAT**	**Actin related protein 3**	**Up**	**168**	**47783**	**5.61**	**15**	**36**	**−**
P85108	TBB2A_RAT	Tubulin beta 2A chain	Up	92	50274	4.78	14	40	+
P48679	LMNA_RAT	Lamin-A	Landmark	401	74564	6.54	48	51	−
Q63610	TPM3_RAT	Tropomyosin-alpha 3 chain	Landmark	202	29217	4.75	18	43	−
P09495	TPM4_RAT	Tropomyosin-alpha 4 chain	Landmark	253	28549	4.66	19	47	−

Regulating proteins									
Q5BKC9	NGEF_RAT	Ephexin-1	Absent	55	81527	5.85	6	9	−
P62142	PP1B_RAT	Serine/threonine-protein phosphatase PP1-beta catalytic subunit	Absent	66	37961	5.84	5	19	−
**O35763**	**MOES_RAT**	**Moesin**	**Absent**	**88**	**67868**	**6.16**	**10**	**16**	**−**
**P14668**	**ANXA5_RAT**	**Annexin A5**	**Up**	**288**	**35779**	**4.93**	**20**	**54**	**−**
**Q68FP1**	**GELS_RAT**	**Gelsolin**	**Up**	**227**	**86413**	**5.76**	**24**	**31**	**−**
O35814	STIP1_RAT	Stress induced phosphoprotein 1	Down	160	63158	6.4	14	22	−
B3GNI6	SEP11_RAT	Septin-11	Down	102	50005	6.24	8	21	−
P38983	RSSA_RAT	40S ribosomal protein SA, laminin receptor 1, LRP/LR; laminin-binding protein precursor p40	Down	51	32917	4.8	4	17	−
P85515	ACTZ_RAT	Alpha-centractin	Down	158	42701	6.19	11	40	−
Q99MZ8	LASP1_RAT	Lim and SH3 domain protein 1	Landmark	126	30351	6.61	9	28	−

Transport proteins									
Q9Z2L0	VDAC1_RAT	Voltage dependent anion selective channel protein 1	Absent	61	30851	8.62	4	19	−
P02793	FRIL1_RAT	Ferritin light chain 1	Absent	140	20793	5.99	11	58	−

Chaperones									
Q5XHZ0	TRAP1_RAT	Heat shock protein 75 kDa, mitochondrial	Absent	118	80639	6.56	12	18	−
P28480	TCPA_RAT	T-complex protein 1 subunit alpha	Absent	115	60835	5.86	9	17	−
P52555	ERP29_RAT	Endoplasmic reticulum protein ERp29	Absent	127	28614	6.23	8	31	−
Q66HD0	ENPL_RAT	Endoplasmin, heat shock protein 90 kDa beta member 1	Up	190	92998	4.72	25	31	−
P06761	GRP78_RAT	78 kDa glucose-regulated protein, heat shock 70 kDa protein 5	Up	307	72473	5.07	38	56	−
P18418	CALR_RAT	Calreticulin	Landmark	192	48137	4.33	18	46	−
P63018	HSP7C_RAT	Heat shock cognate 71 kDa protein	Landmark	157	71055	5.37	22	39	+
P63039	CH60_RAT	60 kDa heat shock protein, mitochondrial	Landmark	200	61088	5.91	20	42	−
P48721	GRP75_RAT	Stress 70 protein	Landmark	161	74097	5.97	25	41	+
Q68FQ0	TCPE_RAT	T-complex protein 1 subunit epsilon	Landmark	119	59955	5.51	18	28	−

Apoptosis									
Q9QZA2	PDC6I-RAT	Programmed cell death 6-interacting protein	Landmark	299	97141	6.15	28	32	−

Enzymes									
Energy metabolism									
P10719	ATPB_RAT	ATP synthase subunit beta, mitochondrial	Absent	146	56318	5.19	24	56	+
P50516	VATA_MOUSE	Vacuolar ATP synthase catalytic subunit A	Up	196	68625	5.42	23	36	−
P62815	VATB2_RAT	Vacuolar ATP synthase subunit B, brain isoform	Up	118	56857	5.57	13	28	–
P13803	ETFA_RAT	Electron transfer flavoprotein subunit alpha, mitochondrial	Landmark	49	35272	8.62	4	13	–
Carbohydrate metabolism									
P11980	KPYM_RAT	Pyruvate kinase isoenzyme M1/M2	Absent	175	58294	6.63	15	25	–
O88989	MDHC_RAT	Malate dehydrogenase, cytoplasmic	Down	69	36631	6.16	5	14	–
P07943	ALDR_RAT	Aldose reductase	Down	103	36230	6.26	10	26	–
P04797	G3P_RAT	Glyceraldehyde-3-phosphate dehydrogenase	Landmark	111	36090	8.14	9	31	–
P04764	ENOA_RAT	Alpha enolase	Landmark	233	47440	6.16	24	58	–
Amino acid metabolism									
P0C2X9	AL4A1_RAT	Delta-1-pyrroline-5-carboxylate dehydrogenase, mitochondrial	Absent	166	62286	7.14	13	25	–
Q01205	ODO2_RAT	Dihydrolipoyllysine-residue succinyltransferase component of 2-oxoglutarate dehydrogenase complex	Absent	84	49236	8.89	9	21	–
Q9JLJ3	AL9A1_RAT	4-trimethylaminobutyraldehyde dehydrogenase	Up	69	54530	6.57	9	21	–
P10860	DHE3_RAT	Glutamate dehydrogenase 1, mitochondrial	Landmark	192	61719	8.05	27	36	–
Fatty acid metabolism									
P35571	GPDM_RAT	Glycerol-3-phosphate dehydrogenase, mitochondrial	Absent	123	81549	6.18	12	19	–
P15650	ACADL_RAT	Long-chain specific acyl-CoA dehydrogenase	Up	90	48242	7.63	10	18	–
Proteins against oxidative stress									
P07632	SODC_RAT	Superoxide dismutase [Cu-Zn]	Absent	134	16073	5.88	8	48	–
Q63081	PDIA6_RAT	Protein disulfide isomerase A6	Absent	109	48542	5	8	23	–
Q8R4A1	ERO1A_RAT	ERO1-like protein alpha	Absent	92	54839	5.83	7	19	–
P35704	PRDX2_RAT	Peroxiredoxin 2	Landmark	91	21941	5.34	7	31	–
P11598	PDIA3_RAT	Protein disulfide isomerase 3	Landmark	320	57044	5.88	33	56	–
P54001	P4HA1_RAT	Prolyl 4-hydroxylase subunit alpha-1	Landmark	237	61202	5.63	20	36	–
Proteasom, ubiquitin system									
Q9JHW0	PSB7_RAT	Proteasome subunit beta type-7	Absent	61	30250	8.13	5	12	–
Q6AXR4	HEXB_RAT	Beta-hexosaminidase subunit beta	Up	92	61888	8.02	11	23	+

Signal transduction									
P62260	1433E_RAT	14-3-3 Protein epsilon	Landmark	127	29326	4.63	13	48	+

Transcription									
Q8VHV7	HNRH1_RAT	Heterogeneous nuclear ribonucleoprotein H	Up	72	49442	5.7	11	28	–
P60123	RUVB1_RAT	RuvB like 1, 49 kDa TATA box-binding protein-interacting protein	Down	88	50524	6.02	6	19	–
Q9JMJ4	PRP19_RAT	Pre-mRNA-processing factor 19	Down	129	55661	6.14	13	28	–

Translation									
Q4KM49	SYYC_RAT	Tyrosyl-tRNA synthetase	Down	175	59420	6.57	16	29	–
P38983	RSSA_RAT	40S ribosomal protein SA, laminin receptor 1, LRP/LR; laminin-binding protein precursor p40	Down	51	32917	4.8	4	17	–
P05197	EF2_RAT	Elongation factor 2	Landmark	135	96192	6.41	18	22	–
Q6P9U8	EIF3H_RAT	Eukaryotic translation initiation factor 3 subunit H	Landmark	132	40051	6.2	10	36	–

Unknown function									
Q4FZT0	STML2_RAT	Stomatin like protein 2	UP	85	38504	8.74	6	21	–

First column: Acc. No. (mascot identification). Second column: entry name (swiss-prot identification). Third column: protein title. Fourth column: expression (categories: absent, up, down, landmark). Fifth column: score (mascot-score). Sixth column: MW (theoretical protein mass [Da]). Seventh column: pI (theoretical isoelectric point). Eighth column: Qm (query matches). Ninth column: Sc (sequence covery [%]). Tenth column: mixed (mixed spot [+/−]).

## References

[1] Lindvall O, Kokaia Z (2009). Prospects of stem cell therapy for replacing dopamine neurons in Parkinson’s disease. *Trends in Pharmacological Sciences*.

[2] Lindvall O, Björklund A (2011). Cell therapeutics in parkinson’s disease. *Neurotherapeutics*.

[3] Pfisterer U, Kirkeby A, Torper O (2011). Direct conversion of human fibroblasts to dopaminergic neurons. *Proceedings of the National Academy of Sciences of the United States of America*.

[4] Martínez-Serrano A, Björklund A (1997). Immortalized neural progenitor cells for CNS gene transfer and repair. *Trends in Neurosciences*.

[5] Durand MM, Chugani DC, Mahmoudi M, Phelps ME (1990). Characterization of neuron-like cell line immortalized from primary rat mesencephalon cultures. *Society for Neuroscience Abstracts*.

[6] Zhong LT, Sarafian T, Kane DJ (1993). bcl-2 inhibits death of central neural cells induced by multiple agents. *Proceedings of the National Academy of Sciences of the United States of America*.

[7] Anton R, Kordower JH, Maidment NT (1994). Neural-targeted gene therapy for rodent and primate hemiparkinsonism. *Experimental Neurology*.

[8] Anton R, Kordower JH, Kane DJ, Markham CH, Bredesen DE (1995). Neural transplantation of cells expressing the anti-apoptotic gene bcl-2. *Cell Transplantation*.

[9] Haas SJP, Wree A (2002). Dopaminergic differentiation of the Nurr1-expressing immortalized mesencephalic cell line CSM14.1 *in vitro*. *Journal of Anatomy*.

[10] Haas SJP, Beckmann S, Petrov S, Andressen C, Wree A, Schmitt O (2007). Transplantation of immortalized mesencephalic progenitors (CSM14.1 cells) into the neonatal Parkinsonian rat caudate putamen. *Journal of Neuroscience Research*.

[11] Hoffrogge R, Beyer S, Hübner R (2007). 2-DE profiling of GDNF overexpression-related proteome changes in differentiating ST14A rat progenitor cells. *Proteomics*.

[12] Winkler C, Fricker RA, Gates MA (1998). Incorporation and glial differentiation of mouse EGF-responsive neural progenitor cells after transplantation into the embryonic rat brain. *Molecular and Cellular Neurosciences*.

[13] Cattaneo E, Conti L (1998). Generation and characterization of embryonic striatal conditionally immortalized ST14A cells. *Journal of Neuroscience Research*.

[14] Gundersen HJG (1978). Estimators of the number of objects per area unbiased by edge effects. *Microscopica Acta*.

[15] Lessner G, Schmitt O, Haas SJP (2010). Differential proteome of the striatum from hemiparkinsonian rats displays vivid structural remodeling processes. *Journal of Proteome Research*.

[16] Vernon PS, Griffin DE (2005). Characterization of an in vitro model of alphavirus infection of immature and mature neurons. *Journal of Virology*.

[17] Eng LF, Ghirnikar RS, Lee YL (2000). Glial Fibrillary Acidic Protein: GFAP-thirty-one years (1969–2000). *Neurochemical Research*.

[18] Svendsen CN, Bhattacharyya A, Tai YT (2001). Neurons from stem cells: preventing an identity crisis. *Nature Reviews Neuroscience*.

[19] Polak JM, van Noorden S (1992). *An Introduction to Immunocytochemistry: Current Techniques and Problems*.

[20] Kermer P, Krajewska M, Zapata JM (2002). Bag1 is a regulator and marker of neuronal differentiation. *Cell Death and Differentiation*.

[21] Haas SJ-P, Petrov S, Kronenberg G, Schmitt O, Wree A (2008). Orthotopic transplantation of immortalized mesencephalic progenitors (CSM14.1 cells) into the substantia nigra of hemiparkinsonian rats induces neuronal differentiation and motoric improvement. *Journal of Anatomy*.

[22] Udenfriend S, Wyngaarden JB (1956). Precursors of adrenal epinephrine and norepinephrine *in vivo*. *Biochimica et Biophysica Acta*.

[23] Levin EY, Levenberg B, Kaufman S (1960). The enzymatic conversion of 3,4-dihydroxyphenylethylamine to norepinephrine. *The Journal of Biological Chemistry*.

[24] McCaffery P, Drager UC (1994). High levels of a retinoic acid-generating dehydrogenase in the meso- telencephalic dopamine system. *Proceedings of the National Academy of Sciences of the United States of America*.

[25] Haque NSK, Leblanc CJ, Isacson O (1997). Differential dissection of the rat E16 ventral mesencephalon and survival and reinnervation of the 6-OHDA-lesioned striatum by a subset of aldehyde dehydrogenase-positive TH neurons. *Cell Transplantation*.

[26] Wallén Å, Zetterström RH, Solomin L, Arvidsson M, Olson L, Perlmann T (1999). Fate of mesencephalic AHD2-expressing dopamine progenitor cells in Nurr1 mutant mice. *Experimental Cell Research*.

[27] Reutelingsperger CPM, Hornstra G, Hemker HC (1985). Isolation and partial purification of a novel anticoagulant from arteries of human umbilical cord. *European Journal of Biochemistry*.

[28] Flower RJ, Rothwell NJ (1994). Lipocortin-1: cellular mechanisms and clinical relevance. *Trends in Pharmacological Sciences*.

[29] Perretti M (1994). Lipocortin-derived peptides. *Biochemical Pharmacology*.

[30] Raynal P, Pollard HB (1994). Annexins: the problem of assessing the biological role for a gene family of multifunctional calcium- and phospholipid-binding proteins. *Biochimica et Biophysica Acta*.

[31] Bonventre JV (1997). Roles of phospholipases A2 in brain cell and tissue injury associated with ischemia and excitotoxicity. *Journal of Lipid Mediators and Cell Signalling*.

[32] Klein J (2000). Membrane breakdown in acute and chronic neurodegeneration: focus on choline-containing phospholipids. *Journal of Neural Transmission*.

[33] Titsworth WL, Liu NK, Xu XM (2008). Role of secretory phospholipase A2 in CNS inflammation: implications in traumatic spinal cord injury. *CNS and Neurological Disorders*.

[34] Moses GSD, Jensen MD, Lue LF (2006). Secretory PLA2-IIA: a new inflammatory factor for Alzheimer’s disease. *Journal of Neuroinflammation*.

[35] Marusic S, Leach MW, Pelker JW (2005). Cytosolic phospholipase A2*α*-deficient mice are resistant to experimental autoimmune encephalomyelitis. *Journal of Experimental Medicine*.

[36] Pinto F, Brenner T, Dan P, Krimsky M, Yedgar S (2003). Extracellular phospholipase A2 inhibitors suppress central nervous system inflammation. *Glia*.

[37] Hayakawa T, Chang MCJ, Rapoport SI, Appel NM (2001). Selective dopamine receptor stimulation differentially affects [3H]arachidonic acid incorporation, a surrogate marker for phospholipase A2-mediated neurotransmitter signal transduction, in a rodent model of Parkinson’s disease. *Journal of Pharmacology and Experimental Therapeutics*.

[38] Tariq M, Khan HA, Moutaery KA, Deeb SA (2001). Protective effect of quinacrine on striatal dopamine levels in 6-OHDA and MPTP models of Parkinsonism in rodents. *Brain Research Bulletin*.

[39] Liu N, Han S, Lu PH, Xu XM (2004). Upregulation of annexins I, II, and V after traumatic spinal cord injury in adult rats. *Journal of Neuroscience Research*.

[40] Reutelingsperger CPM, van Heerde WL (1997). Annexin V, the regulator of phosphatidylserine-catalyzed inflammation and coagulation during apoptosis. *Cellular and Molecular Life Sciences*.

[41] Fadok VA, Voelker DR, Campbell PA, Cohen JJ, Bratton DL, Henson PM (1992). Exposure of phosphatidylserine on the surface of apoptotic lymphocytes triggers specific recognition and removal by macrophages. *The Journal of Immunology*.

[42] Leist M, Jäättelä M (2001). Four deaths and a funeral: from caspases to alternative mechanisms. *Nature Reviews Molecular Cell Biology*.

[43] Zwaal RFA, Schroit AJ (1997). Pathophysiologic implications of membrane phospholipid asymmetry in blood cells. *Blood*.

[44] Takei N, Ohsawa K, Imaia Y, Nakao H, Iwasaki A, Kohsaka S (1994). Neurotrophic effects of annexin V on cultured neurons from embryonic rat brain. *Neuroscience Letters*.

[45] Mizuno H, Asai K, Fujita K (1998). Neurotrophic action of lipocortin 1 derived from astrocytes on cultured rat cortical neurons. *Molecular Brain Research*.

[46] Han S, Zhang K-H, Lu PH, Xu XM (2004). Effects of annexins II and V on survival of neurons and astrocytes *in vitro*. *Acta Pharmacologica Sinica*.

[47] Hatano S, Oosawa F (1966). Isolation and characterization of plasmodium actin. *Biochimica et Biophysica Acta*.

[48] Holmes KC, Popp D, Gebhard W, Kabsch W (1990). Atomic model of the actin filament. *Nature*.

[49] Yin HL, Stossel TP (1979). Control of cytoplasmic actin gel-sol transformation by gelsolin, a calcium-dependent regulatory protein. *Nature*.

[50] Yin HL, Hartwig JH, Maruyama K, Stossel TP (1981). Ca^2+^ control of actin filament length. Effects of macrophage gelsolin on actin polymerization. *Journal of Biological Chemistry*.

[51] Lamb JA, Allen PG, Tuan BY, Janmey PA (1993). Modulation of gelsolin function. Activation at low pH overrides Ca^2+^ requirement. *Journal of Biological Chemistry*.

[52] Robinson RC, Mejillano M, Le VP, Burtnick LD, Yin HL, Choe S (1999). Domain movement in gelsolin: a calcium-activated switch. *Science*.

[53] Burtnick LD, Urosev D, Irobi E, Narayan K, Robinson RC (2004). Structure of the N-terminal half of gelsolin bound to actin: roles in severing, apoptosis and FAF. *The EMBO Journal*.

[54] Jammey PA, Iida K, Yin HL, Stossel TP (1987). Polyphosphoinositide micelles and polyphosphoinositide-containing vesicles dissociate endogenous gelsolin-actin complexes and promote actin assembly from the fast-growing end of actin filaments blocked by gelsolin. *Journal of Biological Chemistry*.

[55] Janmey PA, Stossel TP (1987). Modulation of gelsolin function by phosphatidylinositol 4,5 bisphosphate. *Nature*.

[56] Furnish EJ, Zhou W, Cunningham CC, Kas JA, Schmidt CE (2001). Gelsolin overexpression enhances neurite outgrowth in PC12 cells. *The FEBS Letters*.

[57] Star EN, Kwiatkowski DJ, Murthy VN (2002). Rapid turnover of actin in dendritic spines and its regulation by activity. *Nature Neuroscience*.

[58] Dong JH, Ying GX, Liu X (2006). Lesion-induced gelsolin upregulation in the hippocampus following entorhinal deafferentation. *Hippocampus*.

[59] Mullins RD, Heuser JA, Pollard TD (1998). The interaction of Arp2/3 complex with actin: nucleation, high affinity pointed end capping, and formation of branching networks of filaments. *Proceedings of the National Academy of Sciences of the United States of America*.

[60] Lankes WT, Furthmayr H (1991). Moesin: a member of the protein 4.1-talin-ezrin family of proteins. *Proceedings of the National Academy of Sciences of the United States of America*.

[61] Mangeat P, Roy C, Martin M (1999). ERM proteins in cell adhesion and membrane dynamics. *Trends in Cell Biology*.

[62] Bretscher A, Edwards K, Fehon RG (2002). ERM proteins and merlin: integrators at the cell cortex. *Nature Reviews Molecular Cell Biology*.

[63] Algrain M, Turunen O, Vaheri A, Louvard D, Arpin M (1993). Ezrin contains cytoskeleton and membrane binding domains accounting for its proposed role as a membrane-cytoskeletal linker. *Journal of Cell Biology*.

[64] Turunen O, Wahlström T, Vaheri A (1994). Ezrin has a COOH-terminal actin-binding site that is conserved in the ezrin protein family. *Journal of Cell Biology*.

[65] Henry MD, Agosti CG, Solomon F (1995). Molecular dissection of radixin: distinct and interdependent functions of the amino- and carboxy-terminal domains. *Journal of Cell Biology*.

[66] Pestonjamasp K, Amieva MR, Strassel CP, Nauseef WM, Furthmayr H, Luna EJ (1995). Moesin, ezrin, and p205 are actin-binding proteins associated with neutrophil plasma membranes. *Molecular Biology of the Cell*.

[67] Chishti AH, Kim AC, Marfatia SM (1998). The FERM domain: a unique module involved in the linkage of cytoplasmic proteins to the membrane. *Trends in Biochemical Sciences*.

[68] Hirao M, Sato N, Kondo T (1996). Regulation mechanism of ERM (ezrin/radixin/moesin) protein/plasma membrane association: possible involvement of phosphatidylinositol turnover and rho-dependent signaling pathway. *Journal of Cell Biology*.

[69] Matsui T, Maeda M, Doi Y (1998). Rho-kinase phosphorylates COOH-terminal threonines of ezrin/radixin/moesin (ERM) proteins and regulates their head-to-tail association. *Journal of Cell Biology*.

[70] Zimprich A, Biskup S, Leitner P (2004). Mutations in LRRK2 cause autosomal-dominant parkinsonism with pleomorphic pathology. *Neuron*.

[71] Paisán-Ruíz C, Jain S, Evans EW (2004). Cloning of the gene containing mutations that cause PARK8-linked Parkinson’s disease. *Neuron*.

[72] MacLeod D, Dowman J, Hammond R, Leete T, Inoue K, Abeliovich A (2006). The familial parkinsonism gene LRRK2 regulates neurite process morphology. *Neuron*.

[73] Bradke F, Dotti CG (1999). The role of local actin instability in axon formation. *Science*.

[74] Bradke F, Dotti CG (2000). Changes in membrane trafficking and actin dynamics during axon formation in cultured hippocampal neurons. *Microscopy Research and Technique*.

